# Similarities and Differences in the GFP Movement in the Zygotic and Somatic Embryos of Arabidopsis

**DOI:** 10.3389/fpls.2021.649806

**Published:** 2021-05-28

**Authors:** Kamila Godel-Jędrychowska, Katarzyna Kulińska-Łukaszek, Ewa Kurczyńska

**Affiliations:** Institute of Biology, Biotechnology and Environmental Protection, Faculty of Natural Sciences, The University of Silesia in Katowice, Katowice, Poland

**Keywords:** GFP, plasmodesmata, somatic embryo, symplasmic domain, tissue formation, zygotic embryo

## Abstract

Intercellular signaling during embryo patterning is not well understood and the role of symplasmic communication has been poorly considered. The correlation between the symplasmic domains and the development of the embryo organs/tissues during zygotic embryogenesis has only been described for a few examples, including Arabidopsis. How this process occurs during the development of somatic embryos (SEs) is still unknown. The aim of these studies was to answer the question: do SEs have a restriction in symplasmic transport depending on the developmental stage that is similar to their zygotic counterparts? The studies included an analysis of the GFP distribution pattern as expressed under diverse promoters in zygotic embryos (ZEs) and SEs. The results of the GFP distribution in the ZEs and SEs showed that 1/the symplasmic domains between the embryo organs and tissues in the SEs was similar to those in the ZEs and 2/the restriction in symplasmic transport in the SEs was correlated with the developmental stage and was similar to the one in their zygotic counterparts, however, with the spatio-temporal differences and different PDs SEL value between these two types of embryos.

## Introduction

Intercellular communication and the spatio-temporal regulation of gene expression are global mechanisms that control development. Plants have developed a unique structure, plasmodesmata (PDs), for intercellular communication in which each plant cell can form direct conduits to its neighbors, thus creating domains of cells that share common components. PDs are active channels that control the movement of the factors that regulate plant development (Heinlein, 2002; Sevilem et al., 2015; Otero et al., 2016; Sager and Lee, 2018 and literature therein).

The presence/absence and permeability of PDs lead to the formation of symplasmic domains, e.g., specialized groups of cells that become isolated either due to the absence of PDs or the downregulation of the cytoplasmic flux on the border of the domain (Bayer and Salmon, 2013; Kitagawa and Jackson, 2017 and literature therein). Such transient symplasmic domains may participate in the coordination of plant growth and development (Sager and Lee, 2014).

Why a symplasmic communication survey during plant development is important? What makes PDs an element of the supracellular information exchange system? By identifying which cells and tissues communicate through PDs, it is possible to determine when and where the signaling is related to the developmental processes. Signaling molecules, transcription factors and mRNA can travel through PDs and are thought to influence the developmental processes (Tilsner et al., 2016; Kehr and Kragler, 2018 and literature therein).

Embryogenesis, during which the zygote follows a defined cell division pattern and differentiation to form the mature embryo, is a crucial developmental process in the lives of flowering plants (Schrick and Laux, 2001; Park and Harada, 2008; Smertenko and Bozkhov, 2014 and literature therein). During embryo development, the basic body pattern is established and therefore, understanding the mechanisms that regulate this stage is important because they affect further growth. The details of ZEs development at the morpho-histological and molecular levels have been well described (Capron et al., 2009; Tvorogova and Lutova, 2018). Because the present studies concern an analysis of symplasmic communication/isolation in SE, specifically its correlation with morphogenesis and histogenesis, the differences in the morphology and histology between the ZEs and SEs will be briefly described. The morphological and histological abnormalities in SEs compared to their zygotic counterparts are manifested by an increased number of ground promeristem layers (Levi and Sink, 1991; Mordhorst et al., 1998; Kurczyńska et al., 2007; Jariteh et al., 2015), an abnormal patterning of the root apical meristem (Bassuner et al., 2007), fused cotyledons of the SEs and fused SEs with changes in the cell patterning (Luo and Koop, 1997; Pescador et al., 2008), differences in the embryo size (Tereso et al., 2007; Jin et al., 2014) and malformations of the SEs (Etienne et al., 2013). If the pattern formation is correlated with the determination of organs/tissues during embryogenesis, the question of how symplasmic communication occurs in these embryos arises.

What is known about symplasmic communication during ZEs and SEs development? An analysis of the zygotic embryogenesis of *Capsella bursa-pastoris* (Schulz and Jensen, 1968) and *Torenia fourieri* (Han et al., 2000) showed changes in symplasmic communication from the beginning of ZEs development. Patricia Zambryski’s team conducted fundamental research for determining the correlation between the symplasmic tracer movement and ZE development. It was proven that in *Arabidopsis thaliana*, cell-to-cell communication *via* the PDs conveys positional information that is critical for establishing the axial body pattern during embryogenesis (Kim et al., 2002, 2005b; Burch-Smith and Zambryski, 2010; Burch-Smith et al., 2011). Ruth Stadler’s team conducted another set of studies on Arabidopsis seeds and ZEs. They demonstrated that the establishment of symplasmic domains coincides with the differentiation of specific cells/tissues (Stadler et al., 2005). Changes in symplasmic communication during zygotic embryogenesis were also observed in *Sedum acre* (Wróbel-Marek et al., 2017).

Data concerning the involvement of symplasmic communication/isolation during the development of SEs are scarce. There is much more information about symplasmic communication in explants during the induction phase of embryogenesis than during SEs development (Dubois et al., 1991; Canhoto et al., 1996; Puigderrajols et al., 2001; Verdeil et al., 2001; Grimault et al., 2007; Reis et al., 2008; Godel-Jedrychowska et al., 2020). Because elucidating the patterning mechanisms in embryogenesis requires understanding intercellular communication, a good knowledge of the establishment of the symplasmic domain in embryos of different origins is required. Therefore, the aim of the presented study was to analyze symplasmic communication in the SEs in order to determine whether the symplasmic domains that form in SEs correspond to the developing tissue and organs that is similar to their zygotic counterparts.

## Materials and Methods

### Plant Material and Culture Conditions

The *STM:ER-GFP, STM:1XsGFP, STM:2XsGFP*, and *STM:3XsGFP* transgenic lines were described in Kim et al. (2005a). The *AtGL2:tmGFP9, AtGL2:GFP, AtSUC3:tmGFP9*, and *AtSUC3:GFP* transgenic lines were described in Stadler et al. (2005) and PDBG2OE [PD-located beta 1,3 glucanases that is tagged internally with mCitrine was described in Benitez-Alfonso et al. (2013)]. The seeds of all of the lines were sown into pots with garden soil and vermiculite mixed at a 1:1 volume ratio. The plants were grown under controlled conditions at a temperature of 20–23°C under a 16 h photoperiod with a light intensity of 40 μmol/m^–2^s^–1^ and relative humidity of 60–70%. After 6–8 weeks, siliques with immature zygotic embryos (IZEs) were collected (Gaj, 2001), surface-sterilized for 20 min in a 20% sodium hypochlorite solution and rinsed three times in sterile water. The IZEs were isolated from the seeds in sterile dishes in water using preparation needles under a stereomicroscope. 10–15 IZEs (explants) were grown on a Phytagel solidified (Sigma, Poland; 3.6 g L-1) E5 medium (Sigma, Poland; Gamborg et al., 1968), which had been supplemented with 1.1 mg/ml 2,4-dichlorophenoxyacetic acid (2,4-D, Sigma-Aldrich) and 20 g L^–1^ sucrose (pH 5.8). The embryo culture was conducted at 21°C under a 16h photoperiod at a light intensity of 20 μmol/m^–2^s^–1^ for up to 21 days. Next, SEs at various stages of development were collected. The analyses were repeated three times. The pictures on the plates show the figures that illustrate the representative results for each variant/replication. For the analyses of the ZEs, 45–71 embryos were tested for each line and the number of examined embryos ranged from 18 in the heart stage to 28 in the torpedo stage per one repetition. For the SEs, the total number of embryos that was analyzed was 65 on average and for each developmental stage, it was about 20 per one repetition. The data in the tables are from the documented and collected images that were taken during the study (a range of “n” = the number of embryos per line/stage; Tables 1–4).

**TABLE 1:** Characteristics and comparison of the zygotic and somatic embryos.

	Similarities/differences	
	Zygotic embryo	Somatic embryo
Embryo size	Globular < 100 μm Heart 100 μm Torpedo 300 μm Cotyledonary 700 μm	Globular 100–150 μm Heart 160–250 μm Torpedo 260–400 μm Cotyledonary 410–1000 μm
Morphology	SAM. two cotyledons, radicle.	SAM. sometimes more than two cotyledons, radicle.
Histology	Normal arrangement of tissues in term of the number of cell layers in tissues; protodermis, ground promeristem, provascular tissue.	Tissue arrangement similar to zygotic counterparts, but number of cells within tissue sometimes changed; tissues often built with more layers than zygotic counterparts; protodermis, ground promeristem, provascular tissue.
Symplasmic domains	Relevant to embryo organs and tissues.	Relevant to embryo organs and tissues.
SEL	Between embryo organs; longitudinal arrangement Globular 81 kDa Heart 51 kDa Torpedo 51 kDa Cotyledonary 27 kDa Between embryo tissues; radial arrangement – centripetal Globular 27 kDa Heart 27 kDa Torpedo 27 kDa Cotyledonary 27 kDa Between embryo tissues; radial arrangement – centrifugal Globular 27 kDa Heart 27 kDa Torpedo 27 kDa^2,3^ Cotyledonary 27 kDa	Between embryo organs; longitudinal arrangement Globular 27 kDa Heart 27 kDa Torpedo < 27 kDa Cotyledonary < 27 kDa Between embryo tissues; radial arrangement – centripetal Globular 27 kDa Heart 27 kDa Torpedo 27 kDa* Cotyledonary 27 kDa* Between embryo tissues; radial arrangement – centrifugal Globular 27 kDa Heart 27 kDa^1^ Torpedo 27 kDa*^2,3^ Cotyledonary 27 kDa^1,2^

*Indicates that the exchange of the GFP occurred only between the hypocotyl tissues; ^1^only in the provascular tissue; ^2^in the protodermis and ^3^in the ground promeristem.

**TABLE 2:** Comparison between the ZEs and SEs domains that emerged during development in *Arabidopsis thaliana STM:1XsGFP, STM:2XsGFP*, and *STM:3XsGFP* lines.

	1XsGFP						2XsGFP						3xXsGFP					
Embryo type	ZEs			SEs			ZEs			SEs			ZEs			SEs		
Domain	Globular			Globular			Globular			Globular			Globular			Globular		
% globular embryos	100% (*n* = 18)			100% (*n* = 12)			100% (*n* = 14)			No activity detected (*n* = 15)			100% (*n* = 14)			No activity detected (*n* = 15)		
Comment	No domain was distinguished			No domain was distinguished			No domain was distinguished			Not applicable			No domain was distinguished			Not applicable		
	up to 27 kDa			up to 27 kDa			up to 54 Da						up to 81 kDa					
Domain	Apical	Central	Basal	Apical	Central	Basal	Apical	Central	Basal	Apical	Central	Basal	Apical	Central	Basal	Apical	Central	Basal
% heart embryos	**100% (*n* = 18)**	**100% (*n* = 15)**	**100% (*n* = 17)**	**75% (*n* = 8)**	**96% (*n* = 12)**	**75% (*n* = 10)**	**100% (*n* = 15)**	**100% (*n* = 10)**	**100% (*n* = 19)**	**100% (*n* = 7)**	**100% (*n* = 10)**	**100% (*n* = 7)**	**100% (*n* = 14)**	**100% (*n* = 14)**	**100% (*n* = 15)**	**2% (*n* = 12)**	**86% (*n* = 8)**	**76% (*n* = 12)**
Comment	No domain was distinguished			Three domains: (1) cotyledon node;			No domain was distinguished			Three domains: (1) cotyledon node;			Three domains: (1) cotyledon node;			Two domains: (1) cotyledon node;		
	up to 27 kDa			(2) hypocotyl; (3) root			up to 54 kDa			(2) hypocotyl; (3) root			(2) hypocotyl; (3) root			(2) Hypocotyl/root		
% torpedo embryos	**100% (*n* = 15)**	**100% (*n* = 15)**	**100% (*n* = 18)**	**83% (*n* = 18)**	**86% (*n* = 15)**	**100% (*n* = 15)**	**100% (*n* = 19)**	**100% (*n* = 19)**	**100% (*n* = 18)**	**100% (*n* = 13)**	**75% (*n* = 13)**	**100% (*n* = 17)**	**100% (*n* = 15)**	**100% (*n* = 15)**	**100% (*n* = 13)**	**88% (*n* = 8)**	**79% (*n* = 8)**	**90% (*n* = 15)**
Comment	No domain was distinguished			Three domains: (1) cotyledon node;			Three domains: (1) cotyledon node;			Three domains: (1) cotyledon node;			Three domains: (1) cotyledon node;			Three domains: (1) cotyledon node;		
Comment	up to 27 kDa			(2) hypocotyl; (3) root			(2) hypocotyl; (3) root			(2) Hypocotyl; (3) root			(2) hypocotyl; (3) root			(2) hypocotyl; (3) root		
% cotyledonary embryos	**100% (*n* = 14)**	**100% (*n* = 14)**	**100% (*n* = 16)**	**33% (*n* = 9)**	**85% (*n* = 13)**	**95% (*n* = 11)**	**100% (*n* = 12)**	**100% (*n* = 13)**	**100% (*n* = 19)**	**98% (*n* = 20)**	**96% (*n* = 21)**	**100% (*n* = 21)**	**100% (*n* = 13)**	**100% (*n* = 17)**	**100% (*n* = 15)**	**96% (*n* = 21)**	**100% (*n* = 22)**	**96% (*n* = 21)**
Comment	No domain was distinguished			Three domains: (1) cotyledon node;			Three domains: (1) cotyledon node;			Three domains: (1) cotyledon node;			Three domains: (1) cotyledon node;			Three domains: (1) cotyledon node;		
	up to 27 kDa			(2) hypocotyl; (3) root			(2) hypocotyl; (3) root			(2) hypocotyl; (3) root			(2) hypocotyl; (3) root			(2) hypocotyl; (3) root		

*% – the percentage of embryos that enable GFP movement to the number of embryos tested (according to Kim et al., 2002; Wróbel-Marek et al., 2017). A large% value (bold%) indicates that movement was possible in this stage of embryo.*

**TABLE 3:** Comparison of the movement frequency of the GFP in the SEs and ZEs of the *Arabidopsis thaliana AtGL2:GFP* transgenic line.

Stage of development	Part of embryo/embryo type	Protoderm		Ground promeristem		Provascular tissue	
		ZEs	SEs	ZEs	SEs	ZEs	SEs
Heart	**Apical**	100% (17*/17**)	0% (0*/10**)	100% (18*/18**)	80% (15*/21**)	100% (18*/18**)	0% (0*/10**)
	**Central**	100% (18*/18**)	100% (10*/10**)	100% (18*/18**)	90% (9*/10**)	100% (18*/18**)	0% (0*/10**)
	**Basal**	100% (18*/18**)	100% (10*/10**)	100% (18*/18**)	90% (9*/10**)	100% (18*/18**)	0% (0*/10**)
Torpedo	**Apical**	100% (25*/25**)	95% (18*/19**)	96% (22*/23**)	94% (16*/17**)	96% (24*/25**)	80% (16*/20**)
	**Central**	100% (25*/25**)	95% (20*/21**)	95% (20*/21**)	94% (16*/17**)	95% (20*/21**)	96% (21*/22**)
	**Basal**	100% (25*/25**)	0% (20*/20**)	95% (18*/19**)	5% (1*/20**)	96% (22*/23**)	10% (2*/20**)
Cotyledonary	**Apical**	0% (19*/19**)	93% (15*/16**)	0% (19*/19**)	0% (17*/17**)	0% (19*/19**)	0% (17*/17**)
	**Central**	100% (19*/19**)	100% (17*/17**)	0% (19*/19**)	0% (17*/17**)	0% (19*/19**)	0% (17*/17**)
	**Basal**	100% (19*/19**)	0% (17*/17**)	0% (19*/19**)	0% (17*/17**)	0% (19*/19**)	0% (17*/17**)

*Number of embryos that enabled the movement of GFP; when the analyzed area (apical/central/basal) was filled with GFP above 80%. **Number of embryos tested. The GFP distribution was analyzed in the radial direction and along the apical-basal axis from the areas of the promoter activity. A large% value (**bold%)** indicates that movement was possible in this direction.

**TABLE 4:** Comparison of the movement frequency of the GFP in ZEs and SEs of the *Arabidopsis thaliana AtSUC3:GFP* transgenic line.

		Protoderm		Ground promeristem		Provascular tissue	
Stage of development	Part of embryo/embryo type	ZEs	SEs	ZEs	SEs	ZEs	SEs
Heart	**Apical**	100% (17*/17**)	0% (19*/19**)	100% (17*/17**)	0% (19*/19**)	100% (17*/17**)	0% (19*/19**)
	**Central**	100% (17*/17**)	75% (14*/19**)	100% (17*/17**)	0% (19*/19**)	100% (17*/17**)	0% (19*/19**)
	**Basal**	100% (17*/17**)	95% (19*/20**)	100% (17*/17**)	95% (19*/20**)	95% (18*/20**)	95% (18*/20**)
Torpedo	**Apical**	0% (15*/15**)	95% (18*/19**)	0% (15*/15**)	0% (19*/19**)	0% (15*/15**)	0% (20*/20**)
	**Central**	100% (15*/15**)	96% (21*/22**)	100% (15*/15**)	80% (16*/20**)	100% (15*/15**)	0% (20*/20**)
	**Basal**	100% (15*/15**)	100% (22*/22**)	100% (15*/15**)	95% (19*/20**)	100% (15*/15**)	0% (20*/20**)
Cotyledonary	**Apical**	0% (18*/18**)	90% (18*/20**)	0% (18*/18**)	0% (19*/19**)	89% (16*/18**)	0% (19*/19**)
	**Central**	0% (18*/18**)	96% (19*/20**)	0% (18*/18**)	0% (19*/19**)	0% (18*/18**)	0% (19*/19**)
	**Basal**	100% (18*/18**)	5% (1*/20**)	100% (18*/18**)	0% (19*/19**)	100% (18*/18**)	0% (19*/19**)

*Number of embryos that enabled the movement of GFP; when the analyzed area (apical/central/basal) was filled with GFP in about 80%. **Number of embryos tested. The GFP distribution was analyzed in the radial direction and along the apical-basal axis from the areas of the promoter activity. A large% value (**bold%**) indicates that movement was possible in this direction.

### Histochemical Staining

For the histological analyses, the samples were fixed in a solution of 2.5% (w/v) glutaraldehyde (GA) in a phosphate buffer (pH = 7.0) for 12 h at 4°C. Then, they were embedded in Steedman’s wax as was described in Sala et al. (2019). The sections (5–7 μm thick) were cut using a HYRAX M40 rotary microtome (Zeiss, Oberkochen, Germany) and collected on microscopic slides that were covered with Haupt’s adhesive (according to Barlow and Kurczyńska, 2007). The sections were stained using the periodic acid-Schiff (PAS) reactions and toluidine blue (TBO, Sigma-Aldrich) staining (0.1% water solution of TBO for 5 min).

### Microscopic Observation

In order to analyze the GFP distribution within the ZEs and SEs, serial optical sections of the embryos were obtained using a confocal laser scanning microscope (CLSM; system FLUO-view 1000; Olympus). The GFP was excited using a multi-Argon Laser (laser power 100 mV; Melles Griot BV; Max. 150 mW) at a 488 nm wavelength and an emission at 500–530 nm. Targeted embryos at each stage of development were studied with an objective lens at different magnifications (UPlanFLN 10x-0.30 numerical aperture, UPlanFLN 20x-0.50 numerical aperture, UPlanFLN 40x-1.35 numerical aperture). Observations were also made using an Olympus BX42 epifluorescence microscope equipped with an Olympus XC50 digital camera and software (Nikon, Tokyo, Japan). The GFP was excited at a maximum wavelength of 490 nm [Nikon Plan Fluor 10x objective lens (0.30 numerical aperture); 20x (0.5 numerical aperture); and 40x (0.75 numerical aperture)]. The histological images were acquired with a Nikon Eclipse Ni-U microscope equipped with a Nikon Digital DS-Fi1-U3 camera and software (Nikon, Tokyo, Japan).

### Image Processing

**FIGURE 1:**
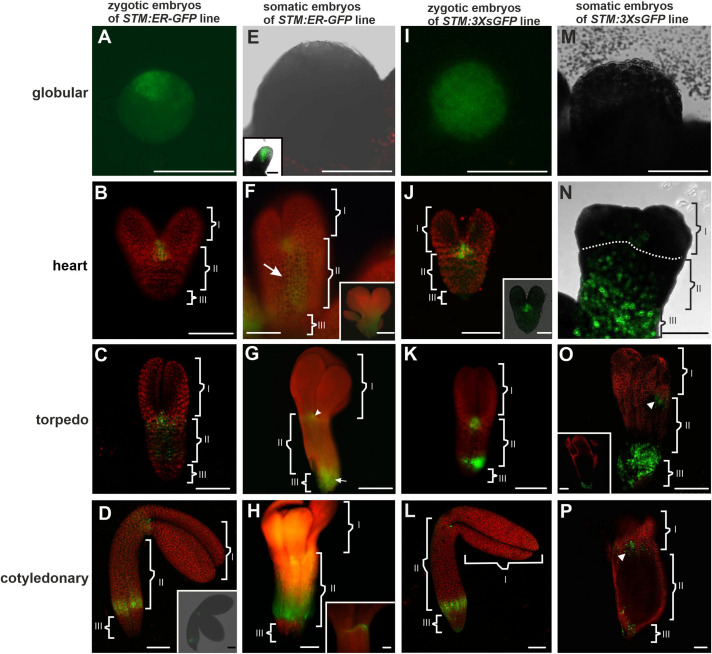
*STM:ER-GFP* promoter activity and localization of the 3XsGFP in the ZEs and SEs. Promoter activity in the **(A)** globular; **(B)** heart, **(C)** torpedo, and **(D)** cotyledonary stages of ZEs. **(D inset)** The optical section of the ZE (from CLSM). **(E)** Globular SE without any visible promoter activity. **(E inset)** Advanced stage of the globular SE with a promoter activity. **(F and F inset)** Heart and **(G)** torpedo stages of the SE. **(H)** Cotyledonary stage of the SE. **(H inset)** The SAM in an embryo in the cotyledonary stage. **(I)** Globular and **(J)** heart stages of the ZEs with the fluorescence of the 3XsGFP. **(J inset)** Optical section through the heart embryo. **(K)** Torpedo and **(L)** cotyledonary stages of the ZE – green fluorescence indicates the presence of the 3XsGFP. **(M)** In the globular SE, the 3XsGFP was not detected. **(N**) Heart and **(O)** torpedo stages of the SE. **(O inset)** The optical section through the basal part of the SE embryo in the torpedo stage. **(P)** Cotyledonary stage of the SE – green fluorescence indicates the presence of the 3XsGFP [arrowheads on **(O,P)** indicate the area with the GFP in the identified SAM area]. The embryo was divided into three parts **I** – apical, **II** – central and **III** – basal. **A,F,F inset,G,H,H inset** – Images from the epifluorescence microscope; **B–D,D inset,J inset,K–O,O inset,P** – images from CLSM. Scale bars; **A,C,D,D inset E,E inset,F,F inset,G–O,O inset**, *P* = 100 μm; **B,J,J inset,H inset** = 50 μm.

Maximum intensity projections (Figures 1B–D,D inset,J inset,K–O,O inset,P, 2D,D inset,F,H,M,O,P, 3B,C,C inset,E,E inset,F,I,I inset,L,L inset, 4E,E inset,F,I,K,L, 5) were created from at least 20 optical sections using FLUOVIEW (Olympus 1.6) and/or ImageJ software. The brightness and contrast of the images that were used for the figure panels were adjusted in Corel Draw X10.

### Classification of the SE Stages

Spherical-shape embryos with an easily distinguishable protodermis and a diameter of about 100 μm were called globular. Heart-shaped, rod-like, or triangular shaped embryos with the cotyledon primordia and size (long axis) between 150 and 250 μm were called the heart. Embryos 250–400 μm long with distinguishable cotyledons were classified as torpedo. The length of the embryos in the cotyledonary stage was greater than 400 μm with a maximum of about 1000 μm (Table 1).

## Results

### Symplasmic Communication Between the Embryo Organs

To compare the symplasmic communication between the embryo organs (along the apical-basal axis) in the *Arabidopsis thaliana* SEs with ZEs, GFP variants of different molecular sizes that were under the control of the *SHOOT MERISTEMLESS* (*STM*) gene promoter were used. In order to trace the mobility of the molecules of 27 kDa (1XsGFP), 54 kDa (2XsGFP), and 81 kDa (3XsGFP), they were compared with the GFP that had been retained in the endoplasmic reticulum (ER-GFP). The analyses concerned: 1/determining the promoter activity sites and 2/determining the distribution of the 1Xs, 2Xs, and 3Xs mobile GFP molecules (sGFP) at various stages of the ZEs and SEs embryo development. The *STM* gene promoter in the ZEs was active in the globular stage (Figure 1A). In the heart (Figure 1B) stage, the gene promoter activity was detected in the shoot apical meristem (SAM) and cells in nearest vicinity. At the torpedo stage (Figure 1C), the area of promoter activity was detected in the cotyledon node and the ectopic expression of promoter activity was observed in some of the cells of the hypocotyl (to facilitate the description of the individual areas of embryos, especially SEs, the following terms were used: apical – comprising the SAM, cotyledon node and cotyledons; central – comprising the hypocotyl and basal – comprising the root pole). In the cotyledonary stage, the *STM* promoter activity was observed in the SAM and in the basal part of the hypocotyl (Figure 1D and inset).

In the SEs in the early globular stage, no *STM* promoter activity was observed (Figure 1E) and this activity appeared in the embryos in the late globular stage (Figure 1E inset). In the heart stage, promoter activity was detected in the SAM and in the hypocotyl (Figure 1F). In the torpedo stage (Figure 1G), the activity of the *STM* promoter was observed in the cells of the emerging SAM (cotyledon node), the hypocotyl and the basal part of the embryo. In the embryos in the cotyledonary stage, a double distribution pattern of the promoter activity was observed: in the SAM cells (Figure 1H inset) and in the basal part of the hypocotyl (Figure 1H). To summarize, the *STM* promoter activity in the heart stage SEs was not only found in the SAM but also in the hypocotyl cells and from the torpedo stage was similar to that described for the ZEs.

The distribution pattern of 3XsGFP, which is expressed under the *STM* promoter in different developmental stages of ZEs and SEs, was also compared. In the globular ZEs, 3XsGFP was distributed almost uniformly in the entire embryo (Figure 1I). In the heart stage embryos, the GFP did not move from the sites of its expression or only moved into the cells in its nearest vicinity (Figure 1J). In the torpedo stage, the GFP distribution pattern in the SAM was similar to the one that was observed for the heart stage, but additionally, the GFP was detected in the basal part of the hypocotyl (Figure 1K). In the cotyledonary stage, the 3XsGFP was detected in the SAM and the basal part of the hypocotyl (Figure 1L). In the globular stage of the SEs, no fluorescence of the 3XsGFP was detected (Figure 1M). In the heart stage, the GFP was detected in the hypocotyl and basal part of the embryo corresponding to novel subdomain(Figure 1N and Table 2). In the torpedo (Figure 1O) and cotyledonary (Figure 1P) stages, the 3XsGFP was present only in the embryo areas that corresponded to the sites of promoter activity. The results suggest that for molecules up to 81 kDa in the ZEs and SEs, three symplasmic domains were present from the torpedo stage (Table 2).

**FIGURE 2:**
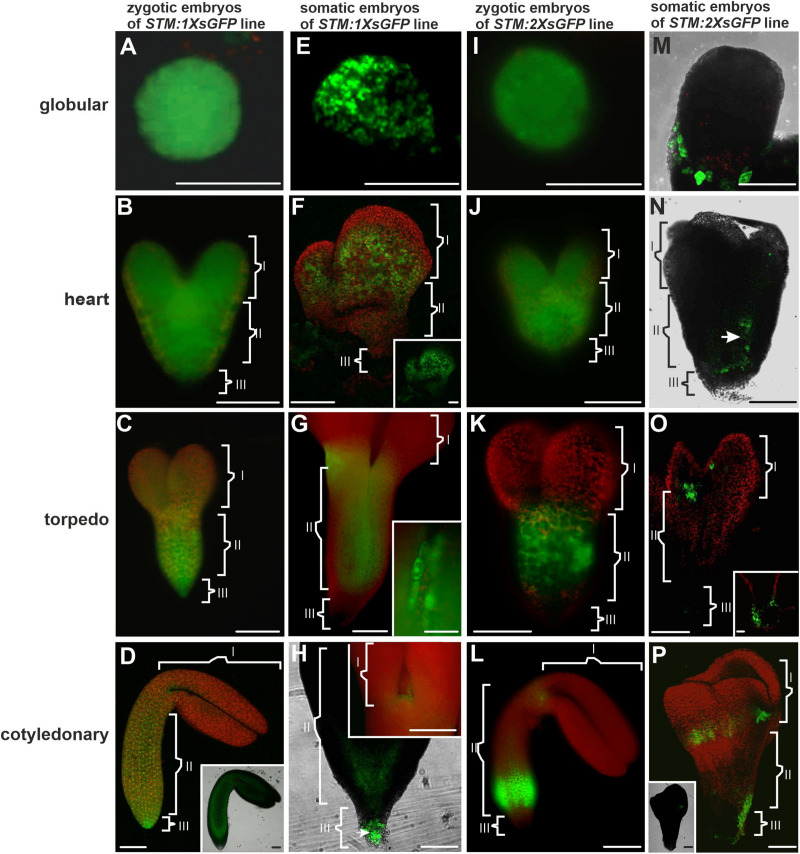
Distribution pattern of the 1XsGFP and 2XsGFP in the ZEs and SEs. Distribution of the 1XsGFP was observed in the entire ZEs at all of the developmental stages: **(A)** globular, **(B)** heart, **(C)** torpedo, and **(D)** cotyledonary. **(D inset)** The optical section (CLSM; only GFP channel) shows the 1XsGFP presence in the entire embryo. **(E)** Globular and **(F)** heart stages of the SE with the fluorescence of the 1XsGFP. **(F inset)** The GFP channel. **(G)** Torpedo stage of the SE. **(G inset)** The intracellular localization of the GFP. **(H)** Embryo in the cotyledonary stage. **(H inset)** The SAM in the embryo in the cotyledonary stage (green fluorescence indicates the presence of the 1XsGFP). **(I)** Globular, **(J)** heart, **(K)** torpedo, and **(L)** cotyledonary stage of the ZEs (green fluorescence indicates the presence of the 2XsGFP). **(M)** In the globular SE, the 2XsGFP was not detected. **(N)** Heart and **(O)** torpedo SE. **(O inset)** Basal part of the torpedo SE. **(P)** Cotyledonary stage of the SE (green fluorescence indicates the presence of the 2XsGFP). **(P inset)** Optical section through the SE in the cotyledonary stage with fluorescence visible in the SAM cells. (**I, II,** and **III** – description is the same as for Figure 1). **A–C,E,G,H inset,I–L** – images from the epifluorescence microscope; **D,D inset, F,H,M,O,P,P inset** – images from the CLSM. Scale bars; **A,C,D,D inset,E–G,H inset,I,K–O,O inset,P,P inset** = 100 μm; **B,J,H** = 50 μm; **G inset** = 10 μm.

The distribution pattern of the 1XsGFP and 2XsGFP was analyzed in both embryo types (Figure 2). The distribution of the 1XsGFP at different stages of the ZEs development showed that all of the domain boundaries permitted the passage of the 1XsGFP to spread from the *STM* expression site (Figures 2A–D). In the SEs in the globular stage, the GFP was detected in the entire embryo (Figure 2E). In the heart stage, the 1XsGFP was present in the entire embryo except for several layers of the cells at the distal parts of the cotyledons and the basal part of the embryo (Figure 2F). This restricted movement of the 1XsGFP in the SEs (Figure 2F) might indicate that novel subdomain boundaries must be established for the movement of the 1XsGFP from the *STM* expression site in the direction toward the distal part of the cotyledons. In the torpedo stage, the 1XsGFP was observed in the hypocotyl and the cotyledon node (Figure 2G). The cotyledonary stage was characterized by the presence of the 1XsGFP only in the SAM, the basal part of hypocotyl and the root (Figure 2H and inset). These results indicate that in SEs, restrictions in symplasmic transport for molecules up to 27 kDa began in the heart stage of embryo development and from the torpedo stage led to the formation of the three symplasmic domains (apical, central and basal, that corresponded to the somatic embryo organs (cotyledon, hypocotyl and root). To summarize: (1) the distribution pattern of the GFP in the ZEs indicates that all of the domain boundaries permitted the passage of molecules up to 27 kDa in all of the developmental stages; for SEs, the distribution pattern of the 1XsGFP indicates the presence of the symplasmic domains and subdomains from the heart stage, and therefore, the domain boundaries had been established earlier than in ZEs; (2) a globular SEs and ZE are a single symplasmic domain in which the SEL of the PDs is at least 27 kDa; (3) in the heart stage SE, the SEL of the PDs between the symplasmic domains is equal to 27 kDa; (4) in the torpedo stage SE, there are three symplasmic domains: a cotyledon and a root meristem domain with the SEL of the PDs equal to or less than 27 kDa and a hypocotyl domain with the PDs SEL on the boundaries that are equal to or more than 27 kDa and (5) in the cotyledonary stage, three symplasmic domain are present (Table 2).

An analysis of the 2XsGFP distribution in the ZEs showed that up to the heart stage, the 2XsGFP was observed throughout the entire embryo (Figures 2I,J). In the torpedo stage, the presence of the 2XsGFP was observed only in the hypocotyl (Figure 2K). This indicates a restriction in the GFP movement into the cotyledons and the basal part of embryos at this stage of development. In the cotyledonary stage, the 2XsGFP was detected in the basal part of the hypocotyl and the SAM (Figure 2L). In the globular stage SEs, no fluorescence of the 2XsGFP was detected (Figure 2M). In the heart stage, the 2XsGFP was observed in groups of irregularly distributed cells in the hypocotyl (Figure 2N). In the SEs in the torpedo stage, the 2XsGFP was detected only in the SAM and root pole (Figure 2O). In the the cotyledonary stage SEs, the 2XsGFP was detected in the cotyledon node cells and in the basal part of the embryo (Figure 2P). To summarize: (1) restrictions in the movement of molecules up to 54 kDa began to occur in the torpedo stage of the ZEs; (2) the 2XsGFP did not move within the SEs to the same extent as it did in the ZEs; (3) the globular ZEs, which comprise one domain as the distribution pattern of 3XsGFP compared with that of ER-GFP, indicate that all of the PD can traffic molecules of at least 81 kDa; (4) in the heart stage, the 2XsGFP appeared to spread into the SE cotyledons from the hypocotyl expression zone (see the ER-GFP pattern in SE); (5) the distribution pattern of the 2XsGFP in the SEs seemed to be more restricted than in the 3XsGFP; (6) the distribution of the 2XsGFP in the torpedo SEs was observed only within the hypocotyl and there was little to no expression in the root pole and (7) in the SE, the 2XsGFP and 3XsGFP did not spread from the location of their expression, unlike in the ZEs.

### Symplasmic Communication Between the Embryo Tissues

The sites of the *AtGL2* promoter activity (*Arabidopsis thaliana GLABRA 2*) were analyzed using the transgenic lines *AtGL2:tmGFP*. The distribution pattern of the GFP molecule between the protodermis and underlying tissues was determined using the *AtGL2:GFP* transgenic line (*AtGL2* promoter/*GFP*; in the *AtGL2:tmGFP* transgenic line, the GFP was fused to the C-terminus of the transmembrane helicase of the AtSTP9 monosaccharide transporter; Stadler et al., 2005).

**FIGURE 3:**
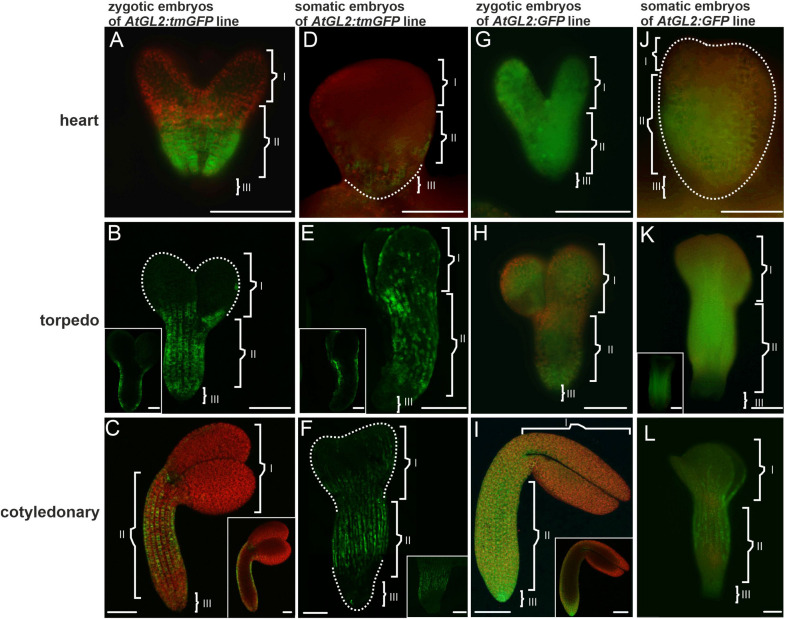
The *AtGL2* promoter activity and GFP distribution pattern in the ZEs and SEs. **(A)** *AtGL2* promoter activity in the ZE in the heart stage, **(B)** in the torpedo and **(C)** cotyledonary stages. **(B,C insets)** An optical section. **(D)** Heart stage of the SE – fluorescence in the central and basal part of the embryo. **(E)** The torpedo stage – promoter activity is present in the protodermal cell of the entire embryo. **(E inset)** An optical section showing the fluorescence in the protodermal cells. **(F)** Cotyledonary stage with fluorescence in the protodermal cells of the hypocotyl and cotyledons. The GFP distribution in the **(G)** heart, **(H)** torpedo, and **(I)** cotyledonary stages of the ZE embryos. **(I inset)** An optical section. The GFP in the **(J)** heart and **(K)** torpedo stages of the SE. **(K inset)** Hand-cut section through the SE – fluorescence in the provascular tissue. **(L)** Cotyledonary stage of the SE. Dotted lines indicate the embryo surface. (**I, II,** and **III** – description is the same as for Figure 1). **A,D,G,H,J,K,K inset** – images from the epifluorescence microscope; **B,C,C inset,E,E inset,F,I,I inset,L,L inset** – images from the CLSM. Scale bars = 100 μm.

The *tmGFP* expression site in the ZEs and SEs indicated that the *AtGL2* promoter was inactive in the globular stage (not shown). It was activated in the heart stage and was expressed in the protodermal cells of the hypocotyl (Figure 3A). These sites of promoter activity persisted in the successive stages of the development of the ZEs and, in some cases, the fluorescence of tmGFP also occurred in the proximal part of the cotyledons (Figure 3B). There was a characteristic pattern in the distribution of the fluorescence of the tmGFP in the hypocotyl in the torpedo stage (Figure 3B inset), which was quite pronounced in the cotyledonary stage (Figure 3C and inset). The protodermal cells in which the tmGFP was expressed formed files along the long embryo axis and alternated with the cells that did not express the tmGFP. The *AtGL2* promoter was only active in the protodermal cells (Figures 3B,C and insets).

In the SEs, promoter activity was mainly observed in the heart stage embryo’s basal parts (Figure 3D). In the torpedo stage, the fluorescence of the tmGFP was mainly observed in the hypocotyl and also in a punctate pattern within the protodermal cells of the cotyledons (Figure 3E). Within the hypocotyl, the *tmGFP*-expressing cells formed irregular files along the organ’s long axis, which alternated with the tmGFP-negative cell files (Figure 3E and inset). The expression of the *tmGFP* in the cotyledonary SEs was detected in the hypocotyl and cotyledons (Figure 3F). Similar to the ZEs, the *AtGL2* promoter was active only in the protodermal cells of the SEs (Figures 3E inset,F inset). The results indicate that there are similarities in the sites of the *AtGL2* promoter activity in the SEs and ZEs, but that in the SEs, the expression pattern of the *tmGFP* in the hypocotyl was quite irregular and was also visible in the cotyledons (Table 3).

An analysis of the GFP distribution in the ZEs of the *AtGL2*:*GFP* line showed that in the heart stage, the GFP was detected throughout the entire embryo in both the protodermis and in the underlying cell layers (Figure 3G). A similar distribution of the GFP was observed for the embryos in the torpedo stage (Figure 3H). In the cotyledonary stage, the GFP fluorescence was only observed in the protodermal cells (Figure 3I and inset). To summarize, the GFP moves until (including) torpedo stage, within the entire embryo from the protodermis to the underlying tissues, thus indicating that the movement of molecules of 27 kDa through the PDs in centripetal direction was possible. In the cotyledonary stage, the protodermis is a symplasmic domain for molecules up to 27 kDa.

An analysis of the GFP distribution in the SEs of the *AtGL2*:*GFP* line, the GFP was not detected in the globular stage (not shown). In the heart stage, the GFP fluorescence was visible throughout the embryo’s hypocotyl and embryo basal parts (Figure 3J). In the torpedo stage, the GFP fluorescence was observed in the hypocotyl protodermis and in the underlying cell layers (Figure 3K inset) as well as in the apical part of the embryo (Figure 3K). In the embryos in the cotyledonary stage, the GFP fluorescence was observed in the hypocotyl and in the proximal and middle parts of the cotyledons. The GFP was not detected in the underlying cell layers (Figure 3L and Table 3). The results indicate that there were restrictions in the symplasmic movement of the GFP between the protodermal cells in the cotyledonary stage in both the SEs and ZEs.

**FIGURE 4:**
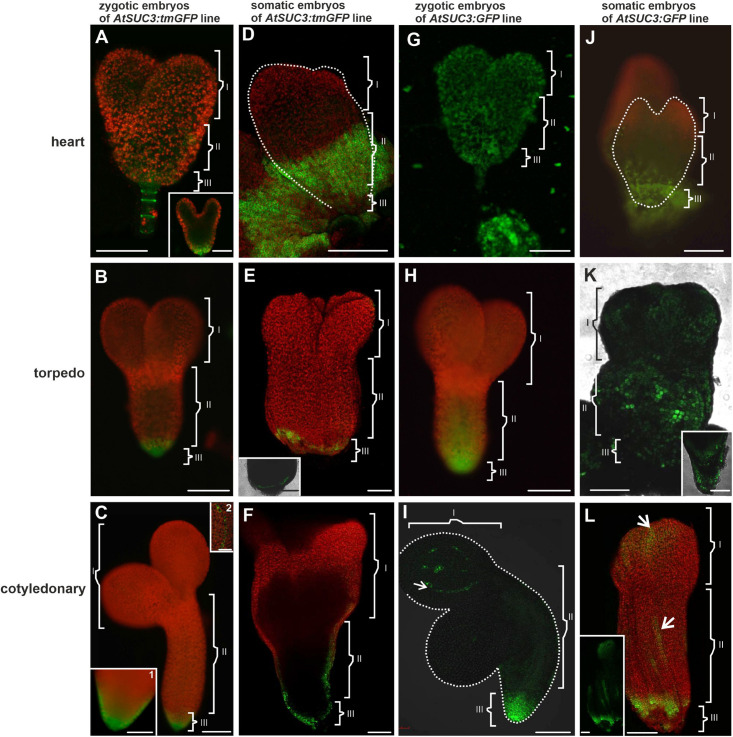
The *AtSUC3* promoter activity and GFP distribution in the ZEs and SEs. The *AtSUC3* promoter was active in the ZE **(A)** in the heart, **(B)** torpedo, and **(C)** cotyledonary stages. In SE: **(D)** in the heart, **(E)** torpedo, and **(F)** cotyledonary stages, the promoter activity was in the central and basal parts of the embryo. The GFP distribution in **(G)** the heart, **(H)** torpedo, and **(I)** cotyledonary stages of the ZE. GFP in **(J)** the heart, **(K)** torpedo, and **(L)** cotyledonary stages of the SE embryos. **(K inset)** An optical section through the basal part of the hypocotyl. **(L inset)** – confocal image showed that the GFP fluorescence was only in the protodermal cells. Dotted lines indicate the embryo surface. (**I, II,** and **III** – description is the same as for Figure 1). **A–C,C insets,D,G,H,J** – images from the epifluorescence microscope; **E,E inset,F,I,K,L** – images from the CLSM. Scale bars: **A,A inset G** – 50 μm; **B,C,C inset 1,D–F,H,I–K,K inset,L,L inset** = 100 μm

The transgenic lines *AtSUC3:tmGFP* and *AtSUC3:GFP* (Arabidopsis *Suc-transporter3 AtSUC3* gene promoter) were used to design these constructs (Stadler et al., 2005). Using the *AtSUC3:tmGFP* line, the sites of the *AtSUC3* promoter activity in the ZEs were examined first. The analysis showed that in the early stages of embryogenesis, the *AtSUC3* promoter was active in the suspensor and the hypophysis (Figure 4A). In the torpedo stage, the promoter activity was observed in all of the cells in the basal part of the embryo (Figure 4B) and in the cotyledonary stage, it was visible in the columella cells and the root cap peripheral cells (Figure 4C, inset 1) as well as in the cotyledon provascular tissue (Figure 4C, inset 2).

In the SEs, the *AtSUC3* promoter was inactive in the globular stage (not shown). In the heart stage, the promoter activity was observed in the cells of the middle (hypocotyl) and basal (the root pole) parts of the embryo (Figure 4D). The area of the tmGFP-derived fluorescence at this stage covered a significant part of the embryo. In the torpedo stage, the tmGFP was expressed only in the cells in the basal part of the embryo (Figure 4E and inset). The expression of the tmGFP in the SEs in the cotyledonary stage was detected in the cells of the root and in the basal part of the hypocotyl (Figure 4F). The results indicate that the sites of the *AtSUC3* promoter activity in the SEs and ZEs are similar and include the embryonic root surface cells; however, in the SEs, especially in the heart stage, the number of cells expressing the *tmGFP* was greater.

The GFP in the ZEs of the *AtSUC3:GFP* line in the heart stage was detected in the cells of the entire embryo (Figure 4G). In the torpedo stage, the GFP fluorescence was observed in the basal and central parts of the embryo, where it was present in the protodermal cells, ground promeristem and provascular tissue (Figure 4H). In the cotyledonary stage, the GFP fluorescence was observed in the basal part of the embryo and in discontinuous cell files (representing the provascular tissue) within the cotyledon (Figure 4I). The results indicate that in the cotyledonary stage of the ZEs, symplasmic isolation occurs between the embryo root and the other embryo organs and between the cells of the provascular tissue and ground promeristem (Table 4).

The presence of the GFP in the SEs of *AtSUC3:GFP* in the heart stage was detected in the central and basal parts of the embryo (Figure 4J). In the torpedo stage (Figure 4K), the GFP fluorescence was seen in the protodermal cells of the entire embryo and in the ground promeristem cells in the basal part of the hypocotyl (Figure 4K and inset). In the cotyledonary stage, the GFP fluorescence was observed throughout the protodermal cells of the hypocotyl and cotyledons but the distribution pattern was patchy (Figure 4L and Table 4).

**FIGURE 5:**
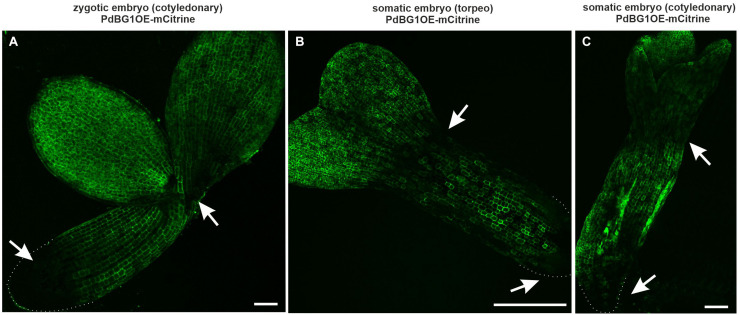
PDGB1 distribution in ZEs and SEs. **(A)** ZE in the cotyledonary stage. **(B)** SE in torpedo and **(C)** in cotyledonary stage. Arrows indicated the areas with lower PDGB1. Dotted lines indicate the embryo root surface. All images are from CLSM. Scale bars; **(A)** = 50 μm; **(B,C)** = 100 μm.

The PdBG1OE-mCitrine line (PdBG1 – a Callose-Degrading Enzyme in PDs; Benitez-Alfonso et al., 2013) was used to determine the involvement of callose in the formation of the symplasmic domains during embryogenesis. This enzyme is directly involved in degrading the β-1,3 glucans and indirectly in modifying the callose deposition in the PDs. The PdBG1 tagged with mCitrine shows areas with a higher enzyme activity that corresponds to less callose deposition (Benitez-Alfonso et al., 2013). Present studies were performed on ZEs in the cotyledonary stage and SEs in the torpedo and cotyledonary stages, for which the symplasmic domains were determined and described above. In the ZEs, the area without the PdBG1 was detected in the basal part of the embryos, which might indicate that there is a higher level of callose compared to the other embryo parts (Figure 5A). In the SEs in torpedo and cotyledonary stages, the areas without the PDBG1 were localized in the cotyledon node and at the boundary between the hypocotyl and the root pole, that is, in the areas that corresponded to the distinguished symplasmic domains (Figures 5B,C).

### Histology of the SE in the Different Developmental Stages

The studies on symplasmic communication in the SEs were accompanied by a histological analysis to define the histology of the SEs. The SE developmental stages were globular (Supplementary Figure 1A and inset), heart (Supplementary Figure 1B), torpedo (Supplementary Figure 1C), and cotyledonary (Supplementary Figure 1D). The embryos had a more or less spherical shape in the globular stage with an easily distinguishable protodermis (Supplementary Figure 1A). Embryos in the heart stage had cotyledon primordia, ground promeristem and provascular tissue (Supplementary Figure 1B and inset). The histological structure of the embryos in the torpedo stage was similar to that of the embryos in the heart stage (Supplementary Figure 1C). The SAM was rarely convex and was usually flat (Supplementary Figure 1C and inset). In the cotyledonary stage, the SEs had well-developed cotyledons, hypocotyl and embryonic root and protodermis, provascular tissue and a ground promeristem (Supplementary Figures 1D,E). The SEs quite often had more than two cotyledons (not shown), fused hypocotyls and roots (Supplementary Figures 1F,H) or had a malformed hypocotyl (Supplementary Figure 1G). The provascular tissue ran from the root meristem along the hypocotyl, then branched and passed into the cotyledons (Supplementary Figure 1E). The abnormalities in tissue arrangement and cytological features of the cells that comprised the tissues were distinct from the cotyledonary stage. The most pronounced malformations were detected in the ground promeristem and provascular strands (Supplementary Figures 1F–H). The files of the ground promeristem cells were not aligned (Supplementary Figure 1D inset) and in many of the SEs were composed of more cell layers than in their zygotic counterparts (Supplementary Figures 1C–H for comparison, a ZE is shown as an inset in Supplementary Figure 1G). The provascular tissue was well visible and like the ground promeristem was composed of more cell files than their zygotic counterparts (Supplementary Figures 1F,H and Table 4).

## Discussion

The establishment of the body pattern during embryogenesis, both zygotic and somatic, is under the control of auxin signaling and differential gene expression (Smertenko and Bozkhov, 2014; Horstman et al., 2017; Fehér, 2019; Tian et al., 2020 and literature therein). Increasing evidence had indicated that symplasmic communication is also involved in the control of embryogenesis (Xu et al., 2012; Brunkard et al., 2015; Choudhary et al., 2020; Godel-Jedrychowska et al., 2020 and literature therein) as well as postembryonic development (Sorkin and Nusinow, 2021; Sager et al., 2021 and literature therein). In the present study, the distribution pattern of the GFP within the SEs and ZEs at different developmental stages was studied to determine the spatio-temporal localization of the symplasmic domains that accompany the establishment of the embryo organs and tissues.

**FIGURE 6:**
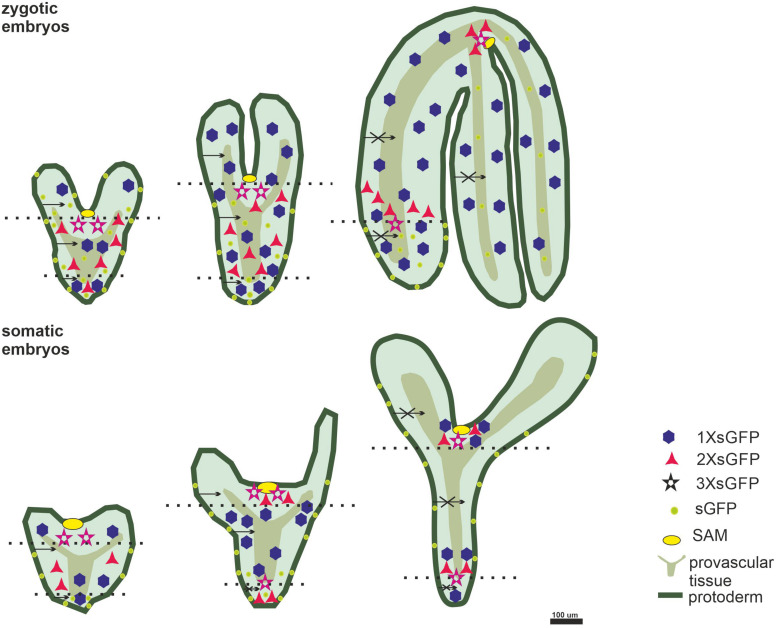
The symplasmic domains identified in the longitudinal direction (between the embryo organs; dotted lines indicate the embryo parts; apical central; basal). In the radial direction – between the embryo tissues; dark green – protodermis; light green – ground promeristem; olive – provascular tissue; octagon – 1XsGFP; triangle – 2XsGFP; star – 3XsGFP; yellow circle – SAM; greenish circle – GFP expressed under *pSUC3*; arrows – the movement in the direction indicated by the arrowhead; a crossed arrows indicates no movement in the direction indicated by the arrowhead.

The results of the GFP distribution in the ZEs and SEs showed that: (1) in the SEs, the symplasmic domains for molecules up to 27 kDa can be distinguished from the heart stage; (2) in the ZEs, the symplasmic domains were established from the torpedo stage for molecules up to 54 kDa; (3) the symplasmic domains between the embryo tissues in the SEs is similar to the one in the ZEs; (4) a key difference between the ZEs and SEs is that in the SE, there is no expression of the STM in the globular stage, which might indicate that the apical-basal polarity is not established at this stage and (5) a restriction in symplasmic transport in the SEs and ZEs is correlated with the developmental stages (Figure 6).

### Symplasmic Domains and Embryo organ Development

During embryogenesis, along the apical-basal axis, the SAM, cotyledons, hypocotyl and radicula are determined and in the radial direction, the protodermis, ground promeristem and provascular tissues are established (Laux et al., 2004). Achieving such an organization requires cell specification in an integrated manner (Laux et al., 2004; Radoeva et al., 2019 and literature therein). The involvement of symplasmic communication/restriction in embryogenesis was first described for development in Arabidopsis ZEs. Studies using the GFP as a mobile fluorescent protein have shown that the symplasmic domains accompany the development of the embryo organs and are established by the mid-torpedo stage with the PDs SEL of 54 kDa at the organ boundaries (Kim et al., 2005a, b). Similar symplasmic domains were detected in the Arabidopsis SEs, but these subdomains appeared in the heart stage and the PDs SEL at their boundaries was determined to be 27 kDa (Figure 6). Regardless of the identified differences, the results support the hypothesis that restrictions in symplasmic communication was correlated with embryo development and the idea that postulates the participation of the PDs as control “points” for the movement of signals during embryogenesis (Otero et al., 2016; Sager and Lee, 2018 and literature therein). The question then arises of whether the identified differences between the ZEs and SEs are developmentally significant. It seems not because the correlation between the emerging domains and the developing embryo organs is clear, and therefore, from a qualitative point of view, there are no differences between the SEs and ZEs in terms of the correlation between the embryo development and the formation of the symplasmic domains. The reason that the limitations in symplasmic communication appear earlier in the SEs than in the ZEs is unknown. It can be presumed that they arise from the morphological heterogeneity (a greater number of cotyledons, the malformation of the SAM and RAM in SEs in comparisons to the ZEs) between the SEs and ZEs that have been described in many species (Dodeman et al., 1997; Mordhorst et al., 1998; Pullman et al., 2003; Etienne et al., 2013; Jariteh et al., 2015) and that was observed in the present study. Such heterogeneity may be the result of disturbances in the spatio-temporal establishment of the apical-basal and radial polarity of the SEs. The detected differences could also have resulted from the diverse capacity of the PDs to transport molecules in these two types of embryos. The GFP movement is (generally) a passive, diffusion-driven transport. Such transport is a function (among others) of the area of passage, the length of the PDs, the wall effects and the electrochemical potential differences between adjacent cells (Liarzi and Epel, 2005; Dashevskaya et al., 2008). It cannot be ruled out that these parameters are different in the ZEs and SEs, at least in the early stages of development. The the shape of the PDs can also influence the GFP movement between organs/tissues (Dashevskaya et al., 2008; Amsbury et al., 2018 and literature therein). Thus, in future studies, the cell wall thickness and the the shape of the PDs in the SEs must also be evaluated.

### Radial Pattering of Embryo and Symplasmic Domains

The results showed that using two transgenic lines, it was possible to trace the GFP distribution pattern between the embryo tissues during embryogenesis. The epidermis has been shown to become symplasmically isolated from the underlying shoot/embryo tissues for tracer dyes and several transcription factors and the reasons for this have previously been discussed (e.g., Roberts and Oparka, 2003; van Bel, 2018). The symplasmic communication between the protodermis and underlying tissues in the ZEs occurred freely in the heart and torpedo stages, thus indicating that these embryos were a single symplasmic domain (Stadler et al., 2005). The results for the SEs were similar to those that were obtained for their zygotic counterparts. In the cotyledonary stage, the protodermis was a distinct symplasmic domain in the ZE and SE, thus indicating that the protodermis, at least for molecules equal to or greater than 27 kDa, was isolated from the underlying tissues. Why is it important to isolate the protodermis as a separate symplasmic domain? Perhaps, this covering tissue must be specified because in the post-embryonic development, it differentiates into several different cell types, but whether this is the only reason is unknown.

Studies on the ZEs of Arabidopsis showed that the embryos in the heart stage that had been derived from the *AtSUC3* promoter/*GFP* plants were a single symplasmic domain (Stadler et al., 2005). In the torpedo stage, only the hypocotyl was a single domain, but in the fully developed embryos, there was restricted movement between the embryo tissues (Stadler et al., 2005). It appeared that in the SEs, the symplasmic domain occurred earlier in the temporal sense but that it was similar to their zygotic counterparts in qualitative sense (Figure 6).

### PD SEL Changes and Embryogenesis

The PDs SEL is regulated during development (Sager and Lee, 2018; Petit et al., 2020 and literature therein) and can be changed by callose deposition (Benitez-Alfonso et al., 2013; Wu et al., 2018; Li et al., 2020 and literature therein). Callose turnover in the PDs plays a key role in different developmental processes (Benitez-Alfonso et al., 2013 and literature therein), including embryogenesis (Du et al., 2020). The reason for the difference in the PDs SEL between the ZEs and SEs is not known. Because the PDs SEL is associated with callose deposition, it seems reasonable to look at this mechanism for an explanation of the detected difference. Results describing the symplasmic communication between the embryonic and non-embryonic areas of an Arabidopsis explant indicated that callose deposition at the PDs is a prerequisite for changing the cell fate (Godel-Jedrychowska et al., 2020). The present results using the PdBG1OE-mCitrine line indicate that callose degradation was lower on the boundaries of the distinguished symplasmic domains along the apical-basal axis. These results support the role of the PDBG1 in callose deposition in the PD and indicate that the establishment of symplasmic domains is important for embryogenesis independent of the origin of an embryo.

## Conclusion

Despite the detected differences in the the spatio-temporal diversity in the formation of the symplasmic domains, there was a clear correlation between the identified domains and the embryo development independent of origin of an embryo (Figure 6). This may indicate that symplasmic communication, which is based on the restrictions of the symplasmic transport of signals, is a mechanism that is involved in regulating embryogenesis.

## Data Availability Statement

The raw data supporting the conclusions of this article will be made available by the authors, without undue reservation.

## Author Contributions

KK-Ł, KG-J, and EK designed the experiments. KK-Ł and KG-J performed the experiments and analyzed the data. EK and KG-J wrote the manuscript. All the authors provided feedback on the manuscript.

## Conflict of Interest

The authors declare that the research was conducted in the absence of any commercial or financial relationships that could be construed as a potential conflict of interest.

## Supplementary Material

Supplementary Figure 1 Histology of the somatic embryos in the different stages of development. **(A)** Somatic embryos in the globular stage (arrow on inset points to the protodermis), **(B,B inset)** heart stage, **(C)** torpedo stage (arrows point to the SAM), **(D,E)** cotyledonary stage. (**D inset**) intercellular spaces between the ground promeristem cells. **(F)** Somatic embryos fused along the root and hypocotyl axis. (**F inset**) vessels on a single cross-section; the appearance of these vessels indicates that the provascular tissue meanders in the embryo. **(G)** A somatic embryo with a malformed hypocotyl. **(G inset)** longitudinal section through the ZE (for comparison to the SE). **(H)** Somatic embryos fused in the hypocotyl part. Staining: 0.05% TBO. Scale bars: **(A)** – 50 μm; **(A inset)** – 20 μm; **(B,C,G inset)** – 100 μm; **(B inset,F inset)** – 50 μm; **(C inset,D inset)** – 30 μm; **(D–H)** – 200 μm.
