# Supplementary figures and images for: Similarities and Differences in the GFP Movement in the Zygotic and Somatic Embryos of Arabidopsis
Source: Front Plant Sci. 2021 May 28;12:649806. doi: 10.3389/fpls.2021.649806 (PMC8194063; doi:10.3389/fpls.2021.649806)

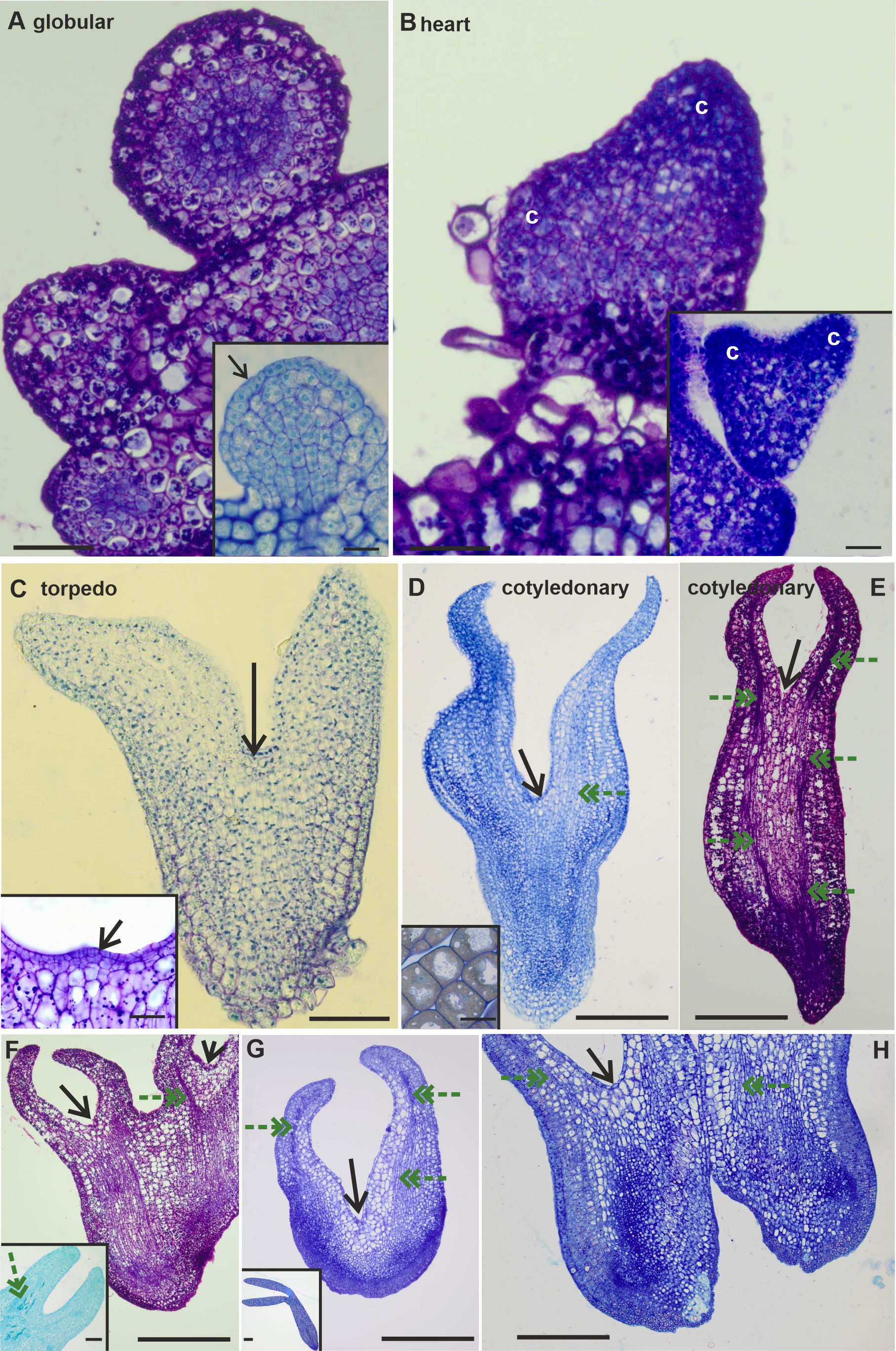

Supplement: Supplementary Figure 1 — Histology of the somatic embryos in the different stages of development. (A) Somatic embryos in the globular stage (arrow on inset points to the protodermis), (B,B inset) heart stage, (C) torpedo stage (arrows point to the SAM), (D,E) cotyledonary stage. (D inset) intercellular spaces between the ground promeristem cells. (F) Somatic embryos fused along the root and hypocotyl axis. (F inset) vessels on a single cross-section; the appearance of these vessels indicates that the provascular tissue meanders in the embryo. (G) A somatic embryo with a malformed hypocotyl. (G inset) longitudinal section through the ZE (for comparison to the SE). (H) Somatic embryos fused in the hypocotyl part. Staining: 0.05% TBO. Scale bars: (A) – 50 μm; (A inset) – 20 μm; (B,C,G inset) – 100 μm; (B inset,F inset) – 50 μm; (C inset,D inset) – 30 μm; (D–H) – 200 μm. [file Image_1.jpeg]
